# Supplementary material for: Combining Brillouin Light Scattering Spectroscopy and Machine-Learned Interatomic Potentials to Probe Mechanical Properties of Metal-Organic Frameworks
Source: J Phys Chem Lett. 2025 Jan 25;16(5):1213–20. doi: 10.1021/acs.jpclett.4c03070 (PMC11808784; doi:10.1021/acs.jpclett.4c03070)
Supplement: Supplementary file 1 — jz4c03070_si_001.pdf [file jz4c03070_si_001.pdf]

Supporting Information:

Combining Brillouin spectroscopy and machine learned interatomic potentials to probe mechanical properties of metal organic frameworks

Florian P. Lindner[1,2,], Nina Strasser[1], Martin Schultze[2], Sandro Wieser[3], Christian Slugovc[4], Kareem Elsayad[5], Kristie J. Koski[6], Egbert Zojer[1], Caterina Czibula[7,*]*

[1]Institute of Solid-State Physics, Graz University of Technology, Petersgasse 16, 8010 Graz, Austria

[2]Institute of Experimental Physics, Graz University of Technology, Petersgasse 16, 8010 Graz, Austria

[3]Institute of Materials Chemistry, TU Wien, Getreidemarkt 9, 1060 Wien, Austria

[4]Institute for Chemistry and Technology of Materials, Graz University of Technology, Stremayrgasse 9, 8010 Graz, Austria

[5]Division of Anatomy, Center for Anatomy and Cell Biology, Medical University of Vienna, Währinger Straße 13, 1090 Vienna, Austria

[6]Department of Chemistry, University of California Davis, 1 Shields Ave. 222 Chemistry, Davis CA, 95616, USA

[7]Institute of Bioproducts and Paper Technology, Graz University of Technology, Inffeldgasse 23, 8010 Graz, Austria

AUTHOR INFORMATION

Corresponding Author

*caterina.czibula@tugraz.at

*florian.lindner@tugraz.at

Contents

1. Details on the experimental methodology 2
2. Details on the theoretical methodology 3
  2.1. VASP simulations and active learning of the MTPs 3
  2.2. Phonon band structures and elastic constants calculated with the MTPs 4
  2.3. Complications encountered when simulating the elastic tensor using VASP in combination with the clamped ion method 5
  2.4. Calculating the elastic tensor using Crystal23 6
  2.5. Calculating the elastic tensor within the cubic approximation 7
3. Additional simulation results augmenting the data shown in the main manuscript 7
  3.1. Calculated elastic tensors 7
  3.2. Longitudinality of the phonon bands 9
4. Details on the Cubic Approximation and Envelope Method 10
5. Going beyond the Cubic Approximation 11
References 13

1. Details on the experimental methodology

In the Brillouin scattering experiments, we used a neodymium yttrium vanadate laser (Coherent Verdi V6) with wavelength 532 nm as light source. The laser power was set to <20 mW. The light was focused onto the sample using a 10X objective (Mitutoyo M Plan Apo SL, NA=0.28, f=200mm) and the (inelastically) scattered laser light was collected by a 20X objective (Mitutoyo M Plan Apo SL, NA=0.28, f=200mm). The collected scattered light was analyzed using a six-pass tandem Fabry-Perot interferometer (JRS Scientific Instruments, TFP-1). The mirror spacing was set to 7 mm and a scan rate of 500 nm was used, allowing a frequency range of the observed Brillouin spectra of about ±20 GHz. The pinholes of the instrument were adjusted to achieve a finesse of approximately 90-100. The schematics of the setup is shown in Figure S1.

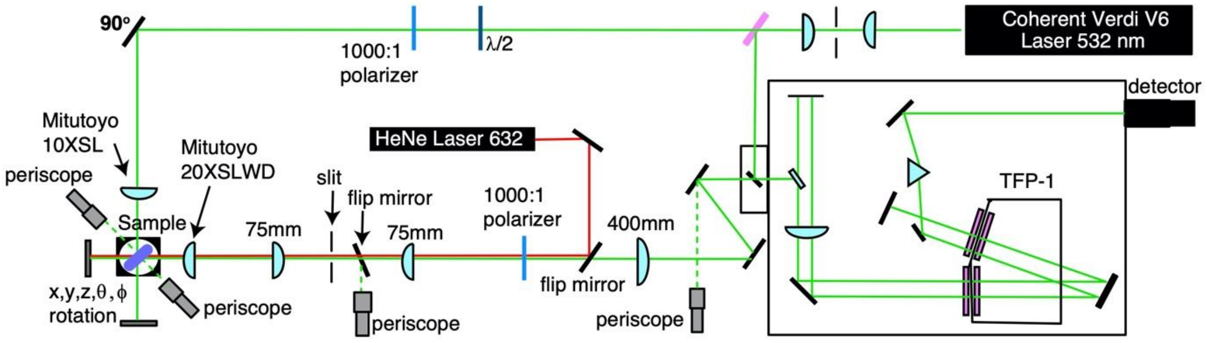

Figure S1. Schematic of the forward-scattering (90a) Brillouin setup used for these measurements. A series of periscopes insures alignment of the laser and sample. The laser used was a Coherent Verdi V6 (λ = 532nm). A 10x Mitutoyo was used for focusing the laser into the sample. A 20X SLWD Mitutoyo with a low numerical aperture (NA = 0.28) was used for light collection. Removable polarizers on the front and back end were adjusted for HH or HV measurements. A λ/2 waveplate is used to rotate the polarization. Spectra were acquired with a tandem scanning Fabry-Perot interferometer (TFP-1).

2. Details on the theoretical methodology

2.1. VASP simulations and active learning of the MTPs

The plane wave cut-off for the VASP[1,2] calculations (version 6.4.1) in this work was set to 800 eV and all calculations were carried out for a 1x1x1 k-grid. The exchange correlation approximation remained at the GGA-level and we used a functional parametrized according to PBE[3,4]. Furthermore, in all our calculations Grimme's D3 dispersion correction[5] with Becke–Johnson damping[6] was applied.

In order to generate DFT reference structures for parametrizing the moment-tensor based force fields (MTPs), used to calculate the elastic tensor, a VASP active-learning-run was performed via an MD run in the NpT ensemble[2,7]. The MD simulation started at 50K and the temperature was gradually increased to 500K in the course of 5000 MD steps with 1fs step size. All other VASP machine learning settings remained at the default values of version 6.4.1. At the start of the procedure, VASP performs several ab-initio MD steps to train a preliminary, kernel-based potential[2], which we refer to as VASP-machine learned potential (VMLP). The so obtained VMLP is used to predict the next step of the MD run. Based on a Bayesian error prediction of the forces between the atoms within the structure of this proposed step, the VASP active-learning algorithm decides whether to perform the VMLP-based MD step, or if the step requires an ab-initio calculation. Any structure calculated using DFT is added to the set of reference structures and the VMLP is retrained on this larger set of sampled structures. As the set of

DFT calculated reference data continues to grow, the VMLP becomes more accurate and fewer DFT steps are required to meet the dynamically adapted Bayesian error threshold criterion. Using the described settings, resulted in 402 reference structures with DFT calculated energies, forces and stresses.

On these reference structures we trained several MTP force fields using MLIP[8]. MTPs describe the energy of a system as a linear combination of fixed basis functions, the name giving moment tensors[9]. The size and accuracy of these basis functions is controlled by a "level" parameter. The level was set to 20. All other parameters remained at their default values. Atom typing was not considered in the setup of the MTPs, i.e., only a single set of parameters was used for each atomic species, independent of their chemical environment. The coefficients of the MTPs were determined by fitting them against the provided DFT data via minimizing a cost function built from energies, forces and stresses (with the respective weights of the different quantities kept at their default values). As the training of MTPs is a stochastic process, in total 5 MTPs were independently trained and we picked the one most suited for the problem at hand.[8,10] In the present case, this was the one yielding no negative frequencies in the calculation of the phonon band structure.

2.2. Phonon band structures and elastic constants calculated with the MTPs

The "best" MTP was then used not only to calculate the elastic tensor of GUT2 (see below), but also to calculate the corresponding phonon band-structure using the phonopy[11,12] package. The atomic displacement distance in phonopy remained at the default value of 0.01 Å. That band structure is shown in **Fehler! Verweisquelle konnte nicht gefunden werden.** of the main manuscript. As in the MTPs all interactions from the DFT calculations are effectively projected onto interactions within a cut-off radius of 5 Å, supercell convergence is reached faster than in simulations using the parent DFT approach. In fact, the extent of the primitive unit cell of GUT2 far exceeds that distance (with unit-cell vector-lengths around 15 Å). Thus, the calculation for that primitive unit cell is converged and does not display any negative phonon modes.

When only group velocities in the long-wavelength limit (i.e., sound velocities) and their angular dependence are considered, these are calculated based on the components of the elastic tensor via the Christoffel dispersion relation and applying the python package "christoffel" by Jaeken et al, version 1.2.1[13].

To calculate the elements of the elastic tensor for the MTPs, we used an in-house script, which applies a stress-strain method. Basically, this script applies 6 different types of strain deformations to the simulation box and extracts the elastic tensor elements C_{ij} directly from Hook's law. In contrast to the VASP calculation discussed below, the positions of the atoms in the distorted structures were fully

relaxed. The deformation size, i.e., the magnitude of the applied strains, is a parameter in the calculation of elastic tensor elements, whose impact needs to be converged. To that end we calculated the elastic constants for various strain sizes, As shown in Figure S2. Except for C_{44} all elements of the elastic tensor only show a minor dependence on the applied strain step-size. The elastic tensor used in the main paper was calculated for a strain size of ~0.6% as in this region of applied strain, C_{44} displayed the smallest variation.

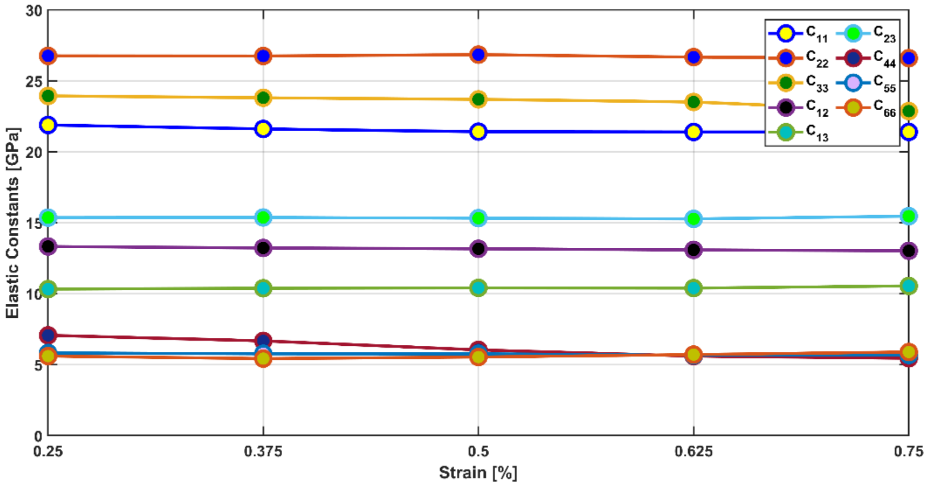

Figure S2. MTP calculated elements of the elastic tensor of GT2 for different strain step sizes. In the calculations a full ionic relaxation of distorted structures was performed.

For calculating averaged engineering constants, the ELATE package (code version 2024.03.15) by Coudert et al.[14] was used.

2.3. Complications encountered when simulating the elastic tensor using VASP in combination with the clamped ion method

When calculating the elements of the elastic tensor employing DFT within the VASP code, as a first step, the geometry was relaxed until the maximum force fell below 10^{-3} eV/Å. A direct calculation of the elastic tensor elements Cij using the VASP codes internal routines[15,16] is difficult and was possible only when employing the so- called clamped-ion method[16]. Here, the positions of the atoms of strained unit cells are not directly relaxed, but the relaxation contribution to the total elastic tensor Cij is only estimated based on the Hessian matrix:

$$\mathbf{C} = \bar{\mathbf{C}} - \frac{1}{V_0}\boldsymbol{\Gamma}^{\mathbf{T}}\mathbf{H}^{-1}\boldsymbol{\Gamma} \quad (1)$$

In equation (1) the matrix $\bar{\mathbf{C}}$ denotes the clamped-ion elastic tensor, this is the elastic tensor obtained from the strained unit cell without allowing the ions to relax to their equilibrium positions again. As $\bar{C}$ would overestimate the true elastic tensor $\mathbf{C}$, it has to be corrected by the second term in eq. (1), the so-called "nuclear relaxation term"[17]. The nuclear relaxation term, comprised from the pseudo-inverse of the Hessian matrix $\mathbf{H}$ and the internal stain tensor $\boldsymbol{\Gamma}$ – for details the interested reader is referred to Wu et al.[16] – corrects $\bar{\mathbf{C}}$ for the effects of the missing ionic relaxation. The cell volume is denoted by V_0. In VASP the so-called POTIM-tag controls both the strain distance in the applied stress-strain method and the displacement amplitude to calculate the 2nd force constants. This is not necessarily ideal. Therefore, we use an in-house developed code, which allowed us to keep the displacement amplitude for the 2nd order force constants at 0.01 Å. Other than that the in-house code performs the same operations as the VASP internal routines to evaluate (1).

However, it proved to be challenging to obtain reliable 2nd order force constants for GUT2, which has a primitive unit cell containing 352 atoms. While our settings (which were at the limit of the computationally feasible) were sufficient to converge the energy to < 1meV/atom, they were apparently not sufficient for converging the 2nd order force constants This was accompanied by negative off-gamma phonon modes in the DFT calculated phonon band structures. A further complication is that the elastic tensor obtained by (1) and the VASP/phonopy 2nd order force constants is nearly singular, with the smallest eigenvalue being in the range of -0.1 GPa up to 0.2 GPa. Moreover, a non-positive definite elastic tensor would also contradict the Born stability criterion for mechanical stability[18]. In passing we note that we encountered similar problems when calculating the elastic properties of more conventional polymeric materials (see discussion in the Supplementary Materials of Reicht et al.[19]).

2.4. Calculating the elastic tensor using Crystal23

In view of these complications, as an alternative, DFT-based approach, elastic constants were calculated using CRYSTAL23[20,21] which is numerically particularly efficient due to the atom-centered basis sets. This is particularly true, when Pople-type basis sets are chosen. As basis sets, we used Zn[8-64111-41G(f)][21–23] for the metallic node and C/N/H/O[6-311G(d,p)][21,23,24] for the linker atoms. This basis set was also used by Tan et al. to calculate elastic constants of ZIF-8 (where it provided the best fit to their experimental values)[23]. The convergence criteria TOLDEG and TOLDEX where set to 0.0002 and 0.0004 respectively. The elastic tensor was calculated for full-ionic-relaxation[20,25] like for the MTPs, which is the default in CRYSTAL[20,21,26]. In the simulations the PBE[3,4] functional was combined with the D3 van der Waals correction[6]. Due to the computational costs of a full strain step size convergence analogous to what has been described above for the MTP, and because of the observed rather minor

dependence of the elastic tensor elements on the strain step size, no systematic tests were performed here. It was decided to calculate the DFT elastic tensor elements for a strain step of 0.05 Å. This strain step is comparable, albeit somewhat smaller than the one applied in the MTP based calculation, which amounts to approx. 0.09 Å (i.e. 0.06% of the unit cell length). For such large strain steps, it was not possible with CRYSTAL23 to find a minimum of the displaced structure within 200 optimization steps even for the first elementary displacement.

Again, for calculating averaged engineering constants, the ELATE package (code version 2024.03.15) by Coudert et al.[14] was applied.

2.5. Calculating the elastic tensor within the cubic approximation

To approximate the elements of the orthorhombic elastic tensor of GUT2, the following equations were used (where the superscript "cub" refers to "cubic" while "orth" referes to orthorhombic.

$$C_{11}^{cub} = \frac{1}{3}\left(C_{11}^{orth} + C_{22}^{orth} + C_{33}^{orth}\right)$$

$$C_{12}^{cub} = \frac{1}{3}\left(C_{12}^{orth} + C_{13}^{orth} + C_{23}^{orth}\right)$$

$$C_{44}^{cub} = \frac{1}{3}\left(C_{44}^{orth} + C_{55}^{orth} + C_{66}^{orth}\right) \qquad (2)$$

3. Additional simulation results augmenting the data shown in the main manuscript

3.1. Calculated elastic tensors

In the following table, the calculated elastic tensors of GUT2 obtained with the best MTPs and with DFT (using CRYSTAL23) are listed.

Table S1: MTP and DFT calculated elastic tensor of GUT 2 for the fully orthorhombic system and within the cubic approximation.

	MTP	DFT
orthorhombic	$C_{\mathrm{MTP}} = \begin{pmatrix} 21.4 & 13.0 & 10.5 & 0 & 0 & 0 \\ 13.0 & 26.6 & 15.5 & 0 & 0 & 0 \\ 10.5 & 15.5 & 22.8 & 0 & 0 & 0 \\ 0 & 0 & 0 & 5.4 & 0 & 0 \\ 0 & 0 & 0 & 0 & 5.7 & 0 \\ 0 & 0 & 0 & 0 & 0 & 5.8 \end{pmatrix} GPa$	$\mathrm{C}_{\mathrm{DFT}} = \begin{pmatrix} 19.8 & 12.4 & 8.7 & 0 & 0 & 0 \\ 12.4 & 22.8 & 14.3 & 0 & 0 & 0 \\ 8.7 & 14.3 & 21.4 & 0 & 0 & 0 \\ 0 & 0 & 0 & 4.4 & 0 & 0 \\ 0 & 0 & 0 & 0 & 5.0 & 0 \\ 0 & 0 & 0 & 0 & 0 & 5.2 \end{pmatrix} \mathrm{GPa}$
cubic approx.	$C_{\mathrm{MTP}} = \begin{pmatrix} 23.6 & 13.0 & 13.0 & 0 & 0 & 0 \\ 13.0 & 23.6 & 13.0 & 0 & 0 & 0 \\ 13.0 & 13.0 & 23.6 & 0 & 0 & 0 \\ 0 & 0 & 0 & 5.7 & 0 & 0 \\ 0 & 0 & 0 & 0 & 5.7 & 0 \\ 0 & 0 & 0 & 0 & 0 & 5.7 \end{pmatrix} GPa$	$\mathrm{C}_{\mathrm{DFT}} = \begin{pmatrix} 21.4 & 11.8 & 11.8 & 0 & 0 & 0 \\ 11.8 & 21.4 & 11.8 & 0 & 0 & 0 \\ 11.8 & 11.8 & 21.4 & 0 & 0 & 0 \\ 0 & 0 & 0 & 4.9 & 0 & 0 \\ 0 & 0 & 0 & 0 & 4.9 & 0 \\ 0 & 0 & 0 & 0 & 0 & 4.9 \end{pmatrix} \mathrm{GPa}$

To conclude this section, we would like to compare the computational resources necessary to obtain the elements of the elastic tensor listed in table S1. Because at first glance it might seem to be a much greater effort to apply the somewhat indirect way of first parametrizing a moment tensor potential than doing a standard ab-initio calculation directly. We assume here that we use the "full-ion-relaxed" method to calculate the elastic tensor, instead of more approximative schemes like the "clamped-ion" method (which shows, as we described above, numerical instabilities for the present case of GUT2). Ultimately our goal is to calculate the second-rank tensor:

$$C_{ij} = \frac{1}{V} \frac{\partial^2 E}{\partial \eta_i \partial \eta_j}$$

In CRYSTAL23 the second derivatives in this equation are calculated numerically by applying strains to the crystal lattice. For each strain, Ns = 3 configurations are defined according to a strain step δ. A symmetry analysis, performed in order to find the minimum set of strains which have to be explicitly applied in order to get all independent elements of C_{ij} yielded six independent strains to be applied. For each strained configuration – with the full-ion-relaxation procedure – the ionic positions have to be relaxed. For a strain step δ of 0.05 a total of 1480 geometry optimization steps were necessary. So, for a structure like GUT2, comprised of 356 atoms, this is quite computationally expensive. On the other hand, we now also review the process to obtain the elements of C_{ij} using MTPs.

As a first step, to parametrize the MTP some sort of DFT based reference structures are necessary. The ability of how well the trained MTPs will be able to describe the MOF system at hand, will depend on how well the provided DFT calculated structures represent the complicated phase-space of the system. As we outlined above in the case of GUT2, within the performed MD-based active learning strategy

during 5000 MD time-steps, with step size 1fs, a total of 402 DFT calculated structures were collected. That is actually all the DFT input that was used to train the MTPs. Compared to the above 1480 DFT steps for the "classical" calculation, the amount of necessary single point DFT steps to prepare the training data for the MTPs is by more than a factor of four smaller.

Of course, now the next step to consider is the training process itself. For the selected type of MTP the training process took around 20h on 2x Xeon Platinum 8174 processors with 24 physical cores, as provided by the Vienna Scientific Cluster 4 (VSC4).

So overall the moment tensor procedure is in fact computationally less expensive than a direct calculation of the elastic tensor elements with the full-ion relaxation procedure.

3.2. Longitudinality of the phonon bands

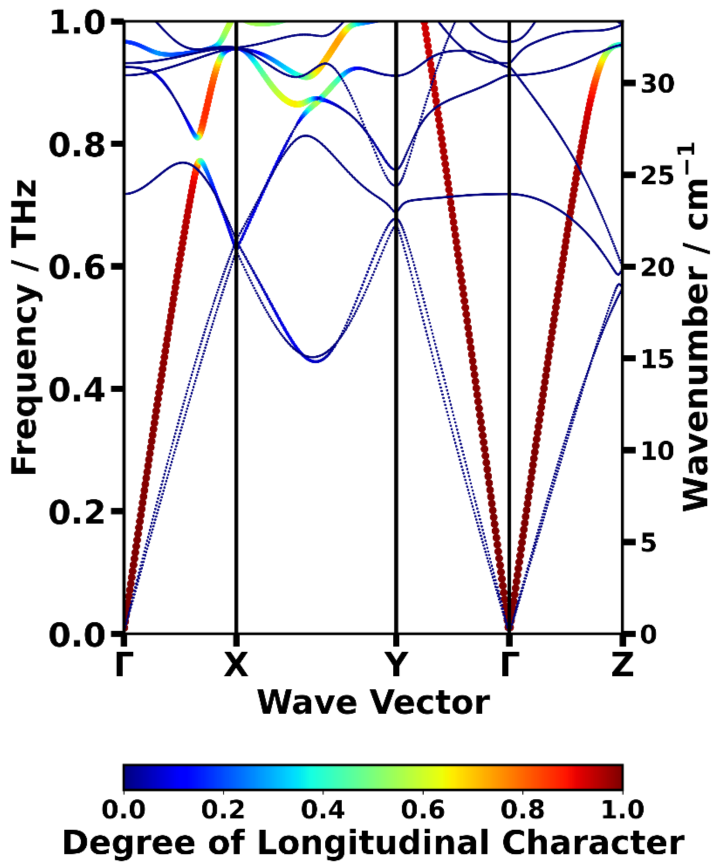

Figure S3.: MTP-calculated low-frequency phonon band structure of GUT2 colored according to the longitudinality of the modes.

To be able to unambiguously distinguish between longitudinal and transverse acoustic modes (at least in high-symmetry directions), Figure S3 shows the phonon band structure of GUT2 colored according

to the longitudinal character of the respective phonons. The longitudinality has been calculated using the formulas described in the work of Strasser et al.[27].

4. Details on the Cubic Approximation and Envelope Method

GUT2 single crystals only reach a size of a few hundred µm and often display a relatively unsmooth surface. In the present case, this, for example, prevents an unambiguous determination of the orientation of the wavevector of the thermally activated phonons involved in the scattering process relative to the crystal axes. This knowledge would, however, be necessary for determining the rather large number of 9 independent non-zero elements of the elastic tensor of the orthorhombic GUT2 crystal. The elastic constants, reveal reasonably similar sets of diagonal (C_{11}, C_{22}, C_{33} as well as C_{44}, C_{55}, and C_{66} and off-diagonal (C_{12}, C_{13}, and C_{23}) elements, see above. This makes the cubic approximation made in the main text appear reasonable. For cubic symmetry, the maxima and minima of the sound velocities are along the high-symmetry <100>, <110>, and <111> directions and one can derive simple expressions relating these extrema to the (only three) independent elastic constants C_{11}, C_{12} and C_{44}. Solving the Christoffel relations along the most relevant directions for transverse and longitudinal acoustic phonons yields the following expressions:

$$(a): v_{QL} = \sqrt{\frac{C_{11}}{\rho}} \text{ along } [1\ 0\ 0] \quad (b): v_{QL} = \sqrt{\frac{C_{11}+2C_{12}+4C_{44}}{3\rho}} \text{ along } [1\ 1\ 1]$$
$$(c): v_{QT} = \sqrt{\frac{C_{44}}{\rho}} \text{ along } [1\ 0\ 0]; \quad (d): v_{QT} = \sqrt{\frac{C_{11}-C_{12}}{2\rho}} \text{ along } [1\ 1\ 0] \qquad (3)$$

Whether the sound velocities in these directions represent the minima and maxima of the measured velocities, depends on the relative magnitudes of the elastic constants (and potentially also on their signs). For the reasons explained above, it is, however, not possible to associate individual experimental datapoints to specific crystallographic directions. Thus, it is crucial, to determine, whether the above equations apply to minima or maxima of the possible sound velocities. To judge the situation in GUT2, the angular dependence of the sound velocities obtained from the cubic (i.e., averaged) elastic constants calculated with the MTPs are shown in Figure S4; the analogous distribution with cubic DFT elastic constants is shown in Figure S5. These distributions suggest in combination with equation (3) that the minimum quasi-longitudinal sound velocity is directly related to the [100] direction and, thus, to C_{11}, that the maximum of v_{QT} determines C_{44}, and that the minimum of v_{QT} is determined by the difference between C_{11} and C_{12}. Based on this analysis, the elastic constants were extracted from the extrema of the sound velocities and are reported in Table1 in the main text. In this context, it should, however, be mentioned that the angular dependent variations of the sound velocities in the cubic approximation are actually very small (much smaller than in the orthorhombic case discussed in the main manuscript).

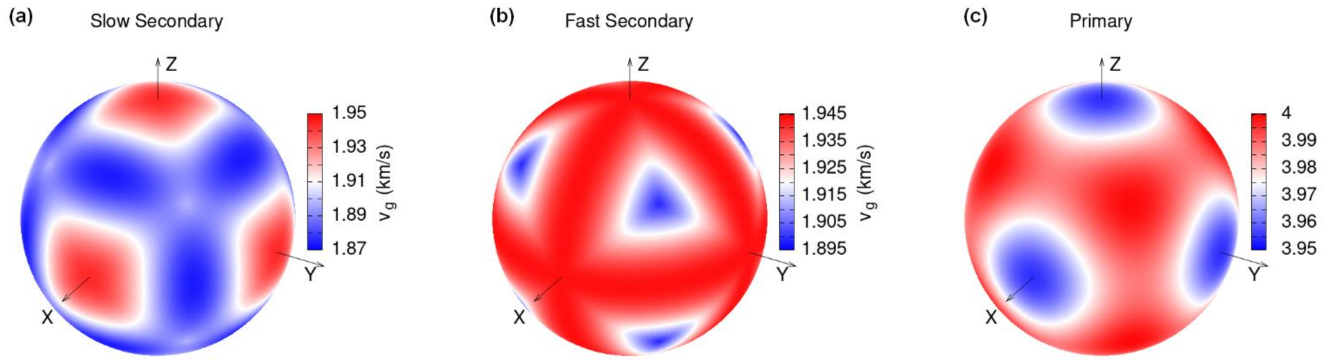

Figure S4. Directional dependence of the MTP-calculated sound velocity distributions in the cubic approximation plotted on a unit sphere. (a) Slow quasi-transversal mode v_{QT2}, (b) fast quasi-transversal mode v_{QT1} and (c) quasi-longitudinal mode v_{QL}.

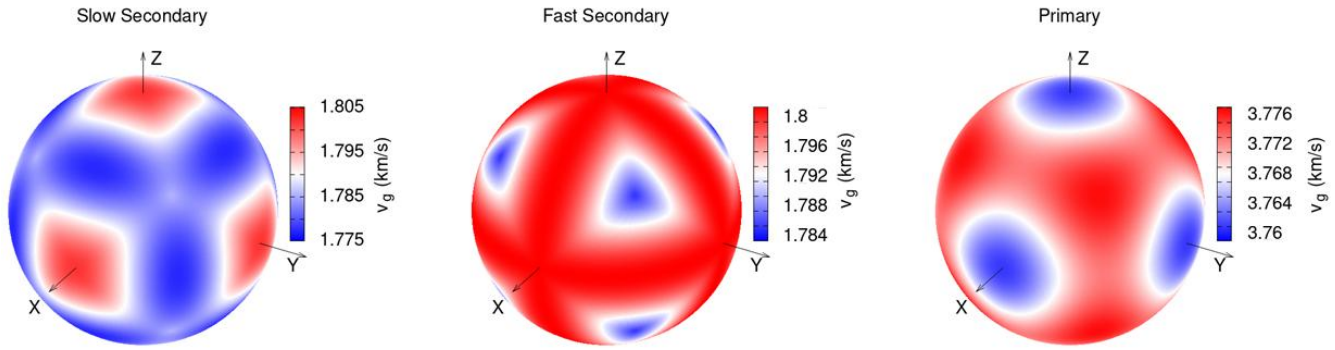

Figure S5. Directional dependence of the DFT-calculated sound velocity distributions in the cubic approximation plotted on a unit sphere.

5. Going beyond the Cubic Approximation

In view of the last statements of the previous section and despite the good agreement between experimentally and theoretically determined elastic constants within the cubic approximation (see main text), it is interesting to assess also the impact of the orthorhombic symmetry of GUT2. In fact, it is not surprising that there are some deviations between the cubic approximation and the full orthorhombic treatment of GUT2 for a quantity as sensitively depending on the elastic constants as the sound velocity. The said MTP-calculated sound velocity distributions for the orthorhombic symmetry are shown in Figure 5 in the main manuscript (MTP calculated) and in Figure S6 (DFT calculated).

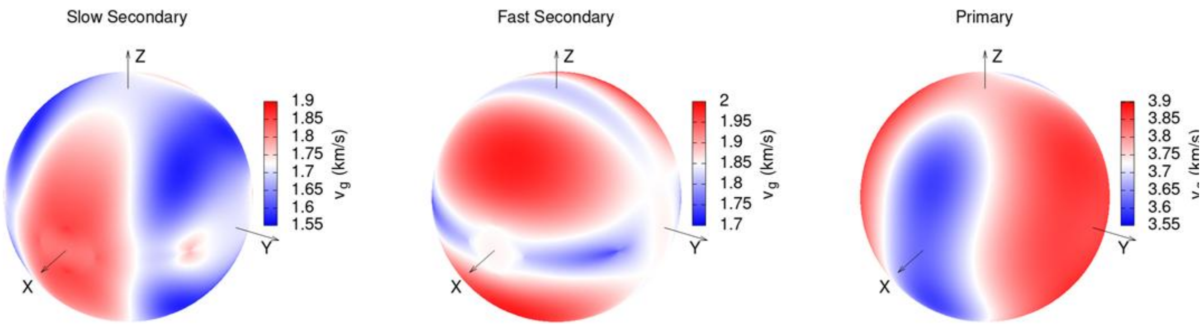

Figure S6 Directional dependence of the DFT-calculated sound velocity distributions for the full-orthorhombic tensor plotted on a unit sphere.

Although, the distribution of the sound velocities for the full orthorhombic elastic tensor deviates from the cubic approximation, the most relevant extrema, namely the maximum of the QL sound velocity and the maxima and minima of the QT sound velocities can still be found in a subset of the directions in which they occurred in the cubic case. One can provide an estimate for the range of the values of the diagonal elements of the full orthorhombic elasticity tensor based on the following observations.

Compared to the cubic case outlined above, the situation changes for the true orthorhombic structure: For q directions parallel to the crystallographic axes, i, the Christoffel equation for the full orthorhombic system yields:

$$v_{QL}^{i} = \sqrt{\frac{C_{ii}}{\rho}} \; with \; i \; \in \{1,2,3\} \quad (4)$$

From the orthorhombic velocity distribution depicted in Figure 5 in the main text and Figure S6, it can be concluded that the extrema of the quasi-longitudinal acoustic velocities actually occur at the crystal axes, except for very minor deviations in the DFT case. Although we do not know the exact crystal orientation during the experiments, and although the sample for technical reasons could be rotated only around a single axis, equation (4) yields an estimate of the minimum and maximum values of C_{11}, C_{22}, and C_{33}. The maximum quasi-longitudinal sound velocity of 4.3 km/s yields an estimate of the upper limit of 27.9 GPa, while the estimate for the lower limit is the same as in the cubic case above. This yields the following scenario: C_{11}, C_{22}, $C_{33} \in$ [18.36 … 27.90] GPa which is consistent with both for the MTP and the DFT simulations. For the quasi-transverse phonons propagating along the directions of the crystallographic axes, an equation similar to eq. (4) holds, albeit now for the shear moduli:

$$v_{QT}^{i} = \sqrt{\frac{C_{ii}}{\rho}}\ with\ i\ \in \{4,5,6\} \quad (5)$$

As in the orthorhombic structure according to Figure 5 and Figure S6 only the maximum value of v_{QT} points in a crystallographic direction, only an upper limit can be provided such that $C_{44}, C_{55}, C_{66} <$ 5.2 GPa. This condition is again fulfilled for the DFT-calculated tensor elements and also in the MTP case they are only slightly higher, by at most 0.6 GPa.

References

1. Kresse, G. & Hafner, J. Ab. initio molecular dynamics for liquid metals. *Phys Rev B* **47**, 558–561 (1992).
2. Jinnouchi, R., Karsai, F., Verdi, C., Asahi, R. & Kresse, G. Descriptors representing two-and three-body atomic distributions and their effects on the accuracy of machine-learned inter-atomic potentials. *J. Chem. Phy.* **152**, 234102 (2020).
3. Perdew, J. P., Burke, K. & Ernzerhof, M. Generalized Gradient Approximation Made Simple. *Phys. Rev. B* **77**, 3865–3868 (1996).
4. Perdew, J. P. *et al.* Erratum: Generalized gredient approximation for the exchange-correlation hole of a many-electron system. *Phys. Rev. B* **5757**, (1998).
5. Grimme, S., Antony, J., Ehrlich, S. & Krieg, H. A consistent and accurate ab initio parametrization of density functional dispersion correction (DFT-D) for the 94 elements H-Pu. *J. Chem. Phys.* **132**, 154104 (2010).
6. Grimme, S., Ehrlich, S. & Goerigk, L. Effect of the damping function in dispersion corrected density functional theory. *J. Comput. Chem.* **32**, 1456–1465 (2011).
7. Jinnouchi, R., Karsai, F. & Kresse, G. On-the-fly machine learning force field generation: Application to melting points. *Phys Rev B* **100**, 014105 (2019).
8. Novikov, I. S., Gubaev, K., Podryabinkin, E. V & Shapeev, A. V. The MLIP package: moment tensor potentials with MPI and active learning. *Mach. Learn.: Sci. Technol.* **2**, 025002 (2021).
9. Shapeev, A. V. Moment tensor potentials: A class of systematically improvable interatomic potentials. *Multiscale Model. Simul.* **14**, 1153–1173 (2016).
10. Wieser, S. & Zojer, E. Machine learned force-fields for an Ab-initio quality description of metal-organic frameworks. *NPJ Comput Mater* **10**, 18 (2024).
11. Togo, A. First-principles Phonon Calculations with Phonopy and Phono3py. *J. Phys. Soc. Jpn.* **92**, 012001 (2023).

12. Togo, A., Chaput, L., Tadano, T. & Tanaka, I. Implementation strategies in phonopy and phono3py. *J. Phys.: Condens. Matter* **35**, 353001 (2023).

13. Jaeken, J. W. & Cottenier, S. Solving the Christoffel equation: Phase and group velocities. *Comput Phys Commun* **207**, 445–451 (2016).

14. Gaillac, R., Pullumbi, P. & Coudert, F. X. ELATE: An open-source online application for analysis and visualization of elastic tensors. *J. Phys.: Condens. Matter* **28**, 275201 (2016).

15. Kresse, G. & Furthmü, J. Efficient iterative schemes for ab initio total-energy calculations using a plane-wave basis set. *Phys Rev B* **54**, 11169–11186 (1996).

16. Wu, X., Vanderbilt, D. & Hamann, D. R. Systematic treatment of displacements, strains, and electric fields in density-functional perturbation theory. *Phys. Rev. B* **72**, 035105 (2005).

17. Jin-Chong Tan. *Mechanical Behaviour of Metal-Organic Framework Materials*. (The Royal Society of Chemistry, 2023).

18. Mouhat, F. & Coudert, F. X. Necessary and sufficient elastic stability conditions in various crystal systems. *Phys Rev B Condens Matter Mater Phys* **90**, (2014).

19. Reicht, L., Legenstein, L., Wieser, S. & Zojer, E. Designing Accurate Moment Tensor Potentials for Phonon-Related Properties of Crystalline Polymers. *Molecules* **29**, (2024).

20. Perger, W. F., Criswell, J., Civalleri, B. & Dovesi, R. Ab-initio calculation of elastic constants of crystalline systems with the CRYSTAL code. *Comput Phys Commun* **180**, 1753–1759 (2009).

21. Dovesi, R. *et al. CRYSTAL23 User's Manual*. (2023).

22. Jaffe, J. E. & Hess, A. C. Hartree-Fock study of phase changes in ZnO at high pressure. *Phys. Rev. B* **48**, 7903–7909 (1993).

23. Tan, J. C. *et al.* Exceptionally low shear modulus in a prototypical imidazole-based metal-organic framework. *PRL* **108**, 095502 (2012).

24. Heyd, J., Peralta, J. E., Scuseria, G. E. & Martin, R. L. Energy band gaps and lattice parameters evaluated with the Heyd-Scuseria-Ernzerhof screened hybrid functional. *Journal of Chemical Physics* **123**, (2005).

25. Erba, A., Mahmoud, A., Orlando, R. & Dovesi, R. Elastic properties of six silicate garnet end members from accurate ab initio simulations. *Phys. Chem. Minerals* **41**, 151–160 (2014).

26. Erba, A. *et al.* CRYSTAL23: A Program for Computational Solid State Physics and Chemistry. *J. Chem. Theory Comput.* vol. 19 6891–6932 Preprint at https://doi.org/10.1021/acs.jctc.2c00958 (2023).

27. Strasser, N., Wieser, S. & Zojer, E. Predicting Spin-Dependent Phonon Band Structures of HKUST-1 Using Density Functional Theory and Machine-Learned Interatomic Potentials. *Int. J. Mol. Sci.* **25**, 3023 (2024).
